# Survival path model outperforms conventional static machine learning models in long-term dynamic prognosis prediction for patients with intermediate stage hepatocellular carcinoma

**DOI:** 10.1093/bioadv/vbaf027

**Published:** 2025-02-17

**Authors:** Lujun Shen, Tao Zhang, Jian Xu, Yiquan Jiang, Fei Cao, Qifeng Chen, Chen Li, Gulijiayina Nuerhashi, Wang Li, Peihong Wu, Weijun Fan

**Affiliations:** Department of Minimally Invasive Therapy, Sun Yat-sen University Cancer Center, Guangzhou 510060, P. R. China; State Key Laboratory of Oncology in South China, Guangdong Provincial Clinical Research Center for Cancer, Sun Yat-sen University Cancer Center, Guangzhou 510060, P. R. China; Department of Information, Nanfang Hospital, Southern Medical University, Guangzhou 510060, P. R. China; Zhongshan School of Medicine, Sun Yat-sen University, Guangzhou 510080, P. R. China; Department of Minimally Invasive Therapy, Sun Yat-sen University Cancer Center, Guangzhou 510060, P. R. China; State Key Laboratory of Oncology in South China, Guangdong Provincial Clinical Research Center for Cancer, Sun Yat-sen University Cancer Center, Guangzhou 510060, P. R. China; Department of Minimally Invasive Therapy, Sun Yat-sen University Cancer Center, Guangzhou 510060, P. R. China; State Key Laboratory of Oncology in South China, Guangdong Provincial Clinical Research Center for Cancer, Sun Yat-sen University Cancer Center, Guangzhou 510060, P. R. China; Department of Minimally Invasive Therapy, Sun Yat-sen University Cancer Center, Guangzhou 510060, P. R. China; State Key Laboratory of Oncology in South China, Guangdong Provincial Clinical Research Center for Cancer, Sun Yat-sen University Cancer Center, Guangzhou 510060, P. R. China; Department of Minimally Invasive Therapy, Sun Yat-sen University Cancer Center, Guangzhou 510060, P. R. China; State Key Laboratory of Oncology in South China, Guangdong Provincial Clinical Research Center for Cancer, Sun Yat-sen University Cancer Center, Guangzhou 510060, P. R. China; Department of Minimally Invasive Therapy, Sun Yat-sen University Cancer Center, Guangzhou 510060, P. R. China; State Key Laboratory of Oncology in South China, Guangdong Provincial Clinical Research Center for Cancer, Sun Yat-sen University Cancer Center, Guangzhou 510060, P. R. China; Department of Minimally Invasive Therapy, Sun Yat-sen University Cancer Center, Guangzhou 510060, P. R. China; State Key Laboratory of Oncology in South China, Guangdong Provincial Clinical Research Center for Cancer, Sun Yat-sen University Cancer Center, Guangzhou 510060, P. R. China; Department of Minimally Invasive Therapy, Sun Yat-sen University Cancer Center, Guangzhou 510060, P. R. China; State Key Laboratory of Oncology in South China, Guangdong Provincial Clinical Research Center for Cancer, Sun Yat-sen University Cancer Center, Guangzhou 510060, P. R. China; Department of Minimally Invasive Therapy, Sun Yat-sen University Cancer Center, Guangzhou 510060, P. R. China; State Key Laboratory of Oncology in South China, Guangdong Provincial Clinical Research Center for Cancer, Sun Yat-sen University Cancer Center, Guangzhou 510060, P. R. China

## Abstract

**Motivation:**

Patients with intermediate stage hepatocellular carcinoma (HCC) require repeated disease monitoring, prognosis assessment, and treatment planning. A novel machine learning model called survival path mapping (SP) model was developed, while its performance as compared with conventional machine learning models remains unknown. Between January 2007 and December 2018, the time-series data of 2644 intermediate stage HCC patients from four medical centers in China were reviewed and included. Static machine learning models by Gaussian Naive Bayes (GNB), support vector machine (SVM), and random forest (RF) for the prediction of survivorship were built based on data at initial admission. Longitudinal data divided into different time slices were utilized for the construction of the SP model. The time-dependent *c*-index was compared between models.

**Results:**

The training set, internal testing set, and external testing set consisted of 1560, 670, and 414 HCC patients, respectively. The survival path model had superior or non-inferior performance in prognosis prediction compared to GNB and RF models since the 12th month after initial diagnosis in the training set and the external testing set. The survival path model had higher time-dependent *c*-index over all conventional ML models since the 6th month in the external testing cohort. In conclusion, the survival path model had superior performance in long-term dynamic prognosis prediction compared to conventional static machine learning models for intermediate stage HCC.

**Availability and implementation:**

The parameters of models are provided in the manuscript.

## 1 Introduction

Hepatocellular carcinoma (HCC) ranks as the sixth most frequently diagnosed type of cancer and is the third most common cause of death from cancer globally (Konyn *et al.* 2021). Patients with intermediate or advanced stage HCC have poor prognosis and always require ongoing monitoring of their condition during treatment. The therapeutic approach for these patients is multifaceted, encompassing interventional procedures, targeted drug therapies, and immunotherapy (Brown *et al.* 2023). The most recent update to the Barcelona Clinic Liver Cancer (BCLC) prognosis and treatment strategy in 2022 incorporated two fresh concepts: treatment stage migration (TSM) and untreatable progression. These additions aim to guide the modification of treatment plans when initial recommendations are suboptimal (Reig *et al.* 2022). In 2023, Alessandro Vitale proposed an evidence-based framework for the treatment of HCC based on the novel concept of “multiparametric therapeutic hierarchy”, which allows dynamic adaption of the staging based algorithms (Vitale *et al.* 2023). Despite these advancements, there remains a significant, unmet need for improved methods of treatments planning that can adapt dynamically to each patient’s changing condition.

In 2018, we introduced a novel analytical approach called survival path mapping, which converts the time-series data into a cascading survival map, in which each survival path bifurcates at fixed time interval depending on the Cox-based selected features (Sonoda *et al.* 2022). The survival path model built in HCC demonstrated to have superior or equal value than conventional staging systems in dynamic prognosis prediction. Meanwhile, many static models based on conventional machine learning algorithms including random forest (RF) (Liao *et al.* 2020), support vector machine (SVM) (Chen *et al.* 2022), and Bayes (Cai *et al.* 2015) had also been developed to compile high throughput data to predict survival of HCC patients. Compared to these traditional static machine learning algorithms, the survival path methodology has advantages in model visualization, ease of understanding, and compatibility with longitudinal data modeling (Shen *et al.* 2023). Currently, the performance of these models in dynamic prognosis prediction for HCC using longitudinal clinical data had not been compared.

In this study, we set out to compare the value of survival path model and traditional static machine learning models in dynamic prognosis prediction for intermediate stage HCC patients. In terms of assessing models’ dynamic predictive ability, Harrell’s *c*-index is not optimal as it overlooks the changes of risk over time (Van Oirbeek and Lesaffre 2010). The time-dependent *c*-index can capture dynamic performance of the regression model over time and provide more precise assessment of models’ discriminative capability (Wang *et al.* 2024) than Harrell’s *c*-index, and therefore is adopted during our analysis.

## 2 Methods

### 2.1 Study design and patient cohorts

**Figure 1.**
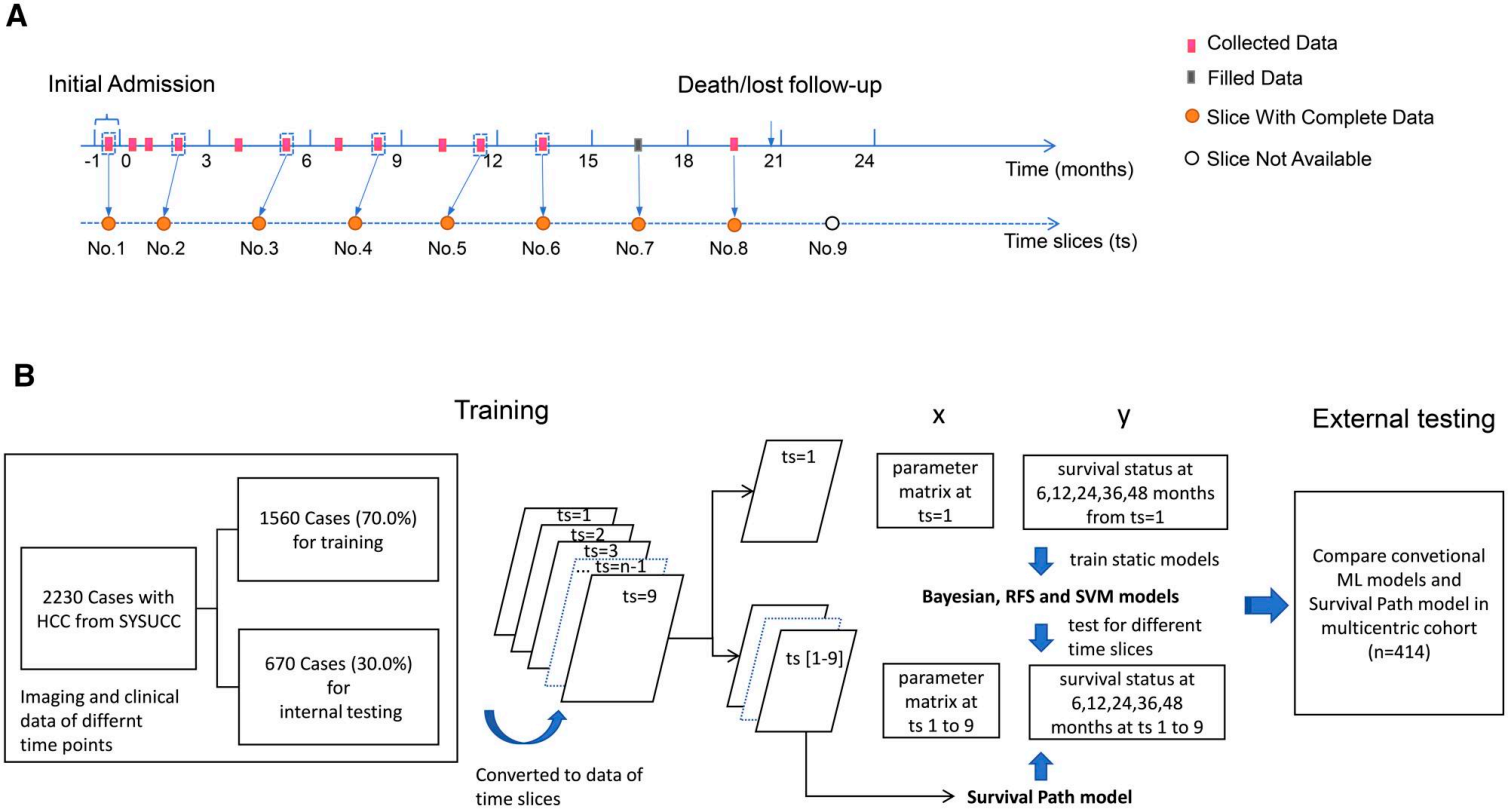
Flowchart of study design. Times-series data of 2230 patients with intermediate stage HCC were included in this study and were separated into training set (*n* = 1560) and internal testing set (*n* = 670), respectively. (A) The time-series data were converted into data of time slices (ts). (B) In building of conventional static ML models, the data of time slice no. 1 were extracted. The conventional models were built based on framework of Bayesian, random forest, or SVM to separately predict survival status of HCC patients at 6, 12, 24, 36, 48 months based on data of time slices no. 1. The construction of survival path model was based on time-series data from ts no. 1 to no. 9. The time-dependent *c*-index of different category of models in prediction of survival based on data of different time slices (ts 1–9) were compared. The results were also validated in a multicentric cohort (*n* = 414).

Between January 2007 and December 2018, 19 582 consecutive patients with newly diagnosed HCC at Sun Yat-sen University Cancer Center (SYSUCC) were retrospectively reviewed as the internal cohort. The inclusion criteria were as follows: (1) clinically or pathologically diagnosed intermediate stage (BCLC stage B) HCC; (2) complete data on intrahepatic tumors at initial diagnosis based on contrast enhanced computed tomography (CT) or magnetic resonance imaging (MRI) of the abdominal region; (3) complete results on radiography or CT of the chest, routine blood test, biochemical routine test, serum AFP level, and coagulation indices during screening period before treatment; (4) without previous or concurrent cancer. A total of 2230 patients were included, which were randomly split into training set (1560, 70%) and internal testing set (670, 30%) (Fig. 1). Public multi-center database of 414 patients with intermediate stage HCC from three medical centers in southern China, which contains times-series clinical data on imaging and blood tests, was utilized as validation cohort. The Hospital Ethics Committee of SYSUCC approved this study, which waived the need for written informed consent based on the retrospective nature of the study.

The majority of HCC patients received transarterial chemoembolization (TACE) based integrated therapies as first-line treatment, which is decided based on the decision of the multidisciplinary teams, including hepatologists, radiologists, and interventional radiologists. The subsequent therapies after failure of first-line treatment included ablation, targeted therapies, and palliative chemotherapy. Patients were advised to receive follow-up monthly during the period of initial treatment, subsequently at every 2–3 months for the first 2 years if complete remission was achieved. The frequency gradually decreased to every 3–6 months after 2 years’ remission.

### 2.2 Transforming of data for analysis

In order to compare the accuracy of different models, patient data, including demographics, imaging, serum tumor markers, and biochemical and coagulation test results, were collected and organized into standard time intervals. For each patient, the starting point was defined as their initial diagnosis of HCC. A 3-month interval was applied, thus transforming the time periods of [−1, 1], [1, 3], [3, 6], [6, 9], [9, 12], [12, 15], [15, 18], [18, 21], [21, 24] months into nine consecutive time slices, indexed from time slice 1 to 9 (Fig. 1A). For variables measured more than once in each time slice, the newest values were selected to be associated with the time slice. The time slice with complete data of the each variable was defined as slice with complete data. If the data in one time slice were incomplete or unavailable, but the follow-up suggested the patient was still alive, the data in this slice and subsequent slices were regarded as point with no available data. For patients who achieved cancer free status (CFS) after treatment and maintain CFS during the late time slices, the missing data of intermediate time slices during monitoring will be filled with the data from previous time slice.

### 2.3 Workflow of survival path mapping

The survival path model is an analytical approach, which converts the time-series data into a cascading survival map, in which each survival path bifurcates at fixed time interval depending on selected key prognostic features by specific (usually Cox-based) learning algorithm. The survival path models were built based on published R package, SurvivalPath (Shen *et al.* 2023). The interval for time slices was set at 3 months and survival path model with nine time slices were computed. The minimum splitting sample size was 15 and the alpha value of significance is set at 0.05. The included variables for survival path model are list in Supplementary Table S1. All the variables included in survival path model can be computed based on variables that included in the construction of conventional machine learning models.

### 2.4 Workflow of conventional static machine learning models

Conventional static machine learning models for predicting overall survival (OS) were generated using the following techniques: Gaussian Naive Bayes (GNB), RF, and SVM. GaussianNB module, RandomForestClassifier module, and SVC module from scikit-learn were utilized for building GNB, RF, and support vector classification (SVC) models, respectively. The data at initial admission (no. 1 time slice) of the derivation cohort served as the parameter matrix and the survivorship at evaluation time of 6, 12, 24, 36, and 48 months, respectively, was set as endpoint in machine learning. The features selected for modeling included age, diameter of hepatic lesions, amount of hepatic lesions, vascular invasion, massive ascites (present/absent), moderate or mild ascites (present/absent), AFP level, ALB level, TBLT level, PT, and Child Pugh score. In the learning and optimization of RF and SVC models, 70% of data serve as training set and 30% serve as the testing set. The predicted results by the three machine learning method for patients of given prediction time, denoted as *t*, and evaluation time, denoted as Δ*t*, were further utilized to calculated time-dependent *c*-index *C*(*t*, Δ*t*) for OS to assess the model’s ability in dynamic prognostication.

### 2.5 Evaluation for prognostic significance

The comparison of prognostic accuracy of different models was conducted in data from time slice no. 1 to no. 9 in the training and internal testing cohort, and time slice no. 1 to no. 6 in the external testing cohort. A total of four models were included and compared in dynamic prognostication for HCC patients, including survival path model, GNB, SVM, and RF. The primary outcome was OS, which was defined as the time from the imaging examination of the interested time slice to death by any causes. The comparison of prognostic significance between survival path model and other conventional machine learning models was conducted in training, internal testing, and external testing datasets (Campani *et al.* 2021), respectively. Time-dependent *c*-index *C*(*t*, Δ*t*) of different models/staging systems was compared to assess their ability in dynamic prognostication, where *t* indicates the prediction time, which is the time when the prediction is made to incorporate dynamic predictions, and Δ*t* denotes the evaluation time, which is the time elapsed since the prediction is made.

### 2.6 Statistical analysis

Pearson $\chi^{2}$ test was used to compare categorical variables between groups. To compare the efficacy in dynamic prognosis prediction between different models, the measurements of time-dependent *c*-index of specific prediction time (*t*) and evaluation time (Δ*t*) were computed using pec R package; means and standard deviations were obtained via 10 random sampling of two-thirds of cases. For category prediction models, subgroups less than three cases were omitted to reduce inference from extreme cases when computing time-dependent *c*-index. A random seed was set using the base R package. The comparison of time-dependent *c*-index between different models was conducted using *Z* test method. A total of 55 tests were conducted during the comparison of time-dependent *c*-index between models. To avoid false positives caused by multiple tests, a meaningful alpha value is set at 0.0009 (0.05/55). All analyses were done using R 3.6.3 (The R Foundation for Statistical Computing 2020).

## 3 Results

### 3.1 The baseline characteristic of the patients

The training set consisted of 1560 HCC patients, the internal testing set consists of 670 HCC patients and the external testing set consisted of 414 HCC patients (Table 1). The median follow-up time were 39.5, 41.0, and 46.0 months for the training set, internal testing set, and external testing set, respectively. Compared to the training set, the internal testing set had a lower proportion of patients with HBV infection (90.3% versus 92.8%; *P* = .050), the external testing set had a higher proportion of patients with younger age (age < 50; 45.4% versus 36.5%; *P* = .001), female gender (15.9% versus 10.6%; *P* = .003), and HBV infection (96.4% versus 92.8%; *P* = .008).

**Table 1.** Baseline characteristics of training set, internal testing set, and multi-center external testing set at initial diagnosis.

Characteristics	Training set (*n* = 1560)		Internal testing set (*n* = 670)		*P*-value	External testing set (*n* = 414)		*P*-value
	No.	%	No.	%		No.	%	
Age (years)					.318			.001
<50	570	36.5	230	34.3		188	45.4	
≥50	990	63.5	440	65.7		226	54.6	
Gender					.593			.003
Male	1395	89.4	594	88.7		348	84.1	
Female	165	10.6	76	11.3		66	15.9	
HBV infection					.050			.008
Absent	113	7.2	65	9.7		15	3.6	
Present	1447	92.8	605	90.3		399	96.4	
AFP (IU/ml)					.951			.266
<25	505	32.4	216	32.2		146	35.3	
≥25	1055	67.6	454	67.8		268	64.7	
Child Pugh Class					.275			.080
A	1433	91.9	606	90.4		369	89.1	
B	127	8.1	64	9.6		45	10.9	
Tumor size (cm)					.301			.702
<5	512	32.8	235	35.1		140	33.8	
≥5	1048	67.2	435	64.9		274	66.2	
Number of lesions					.814			.445
<4	658	42.2	279	41.6		166	40.1	
≥4	902	57.8	391	58.4		248	59.9	

### 3.2 Correlation between covariates and survival outcome

**Figure 2.**
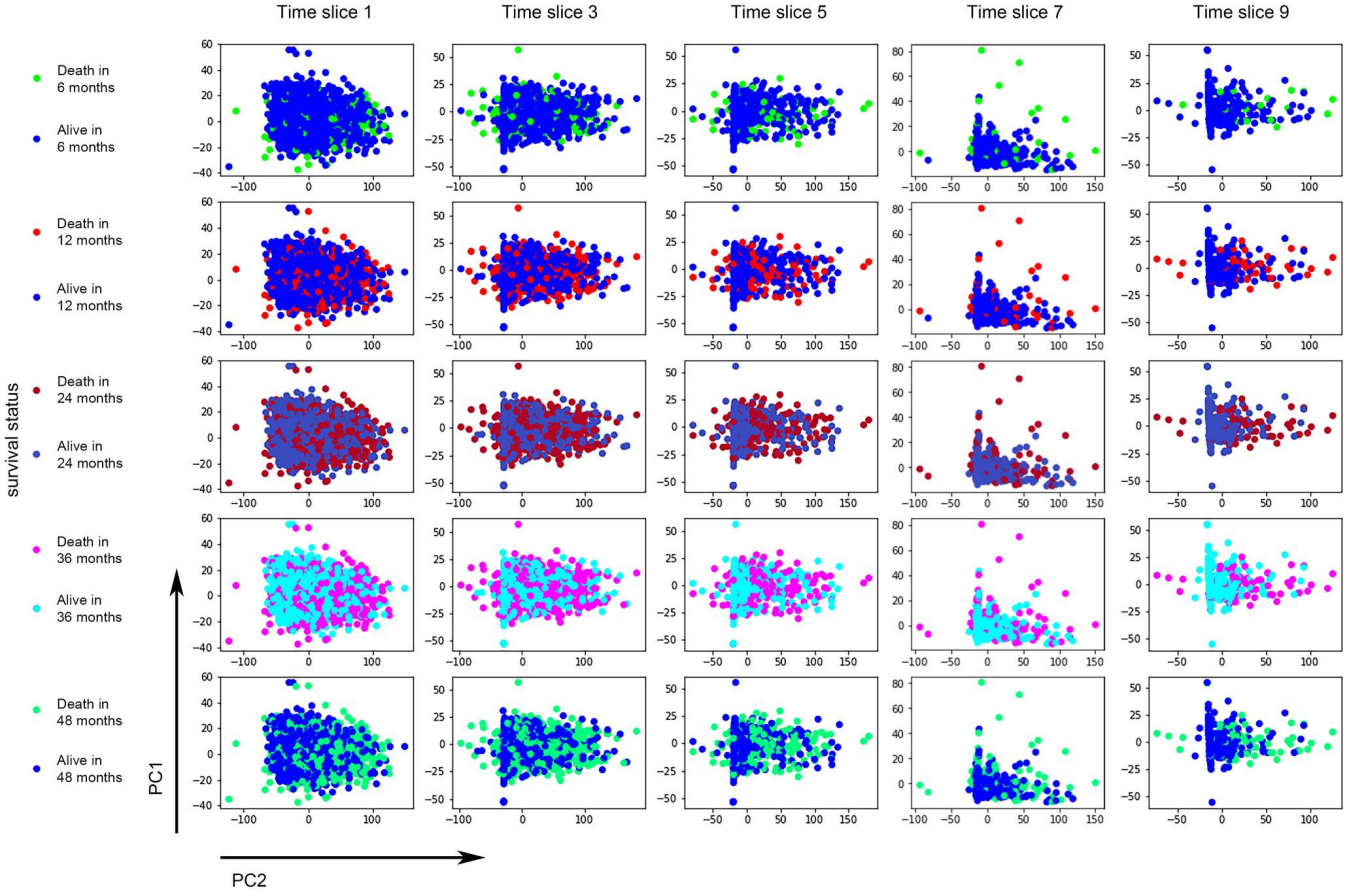
Scatter plot of the correlation between the survival outcome and key features/variables using PCA. The scatter plot describes the correlations between the survival outcome and 16 key features/variables of intermediate-stage liver after dimensionality reduction using PCA. In all subgraphs, the data points for “death” and “survival” overlap, suggesting that using variables from a single time point alone is insufficient to accurately predict whether a patient will have died at a specific time point. An outlier point with PC1 more than two times of the maximal value of the rest patients in PC1 at time slice no. 7 was removed to gain a better visualization of the relationship between the distribution of points and the outcomes.

We first explored the relationship between included key variables and survival outcomes at specific time points in the training set. After dimensionality reduction using PCA, the data points in each subgraph for “death” and “survival” present to be overlapped (Fig. 2), indicating that it is insufficient to accurately predict patient outcome at a specific time point using variables from a single time point alone.

### 3.3 The survival paths model

**Figure 3.**
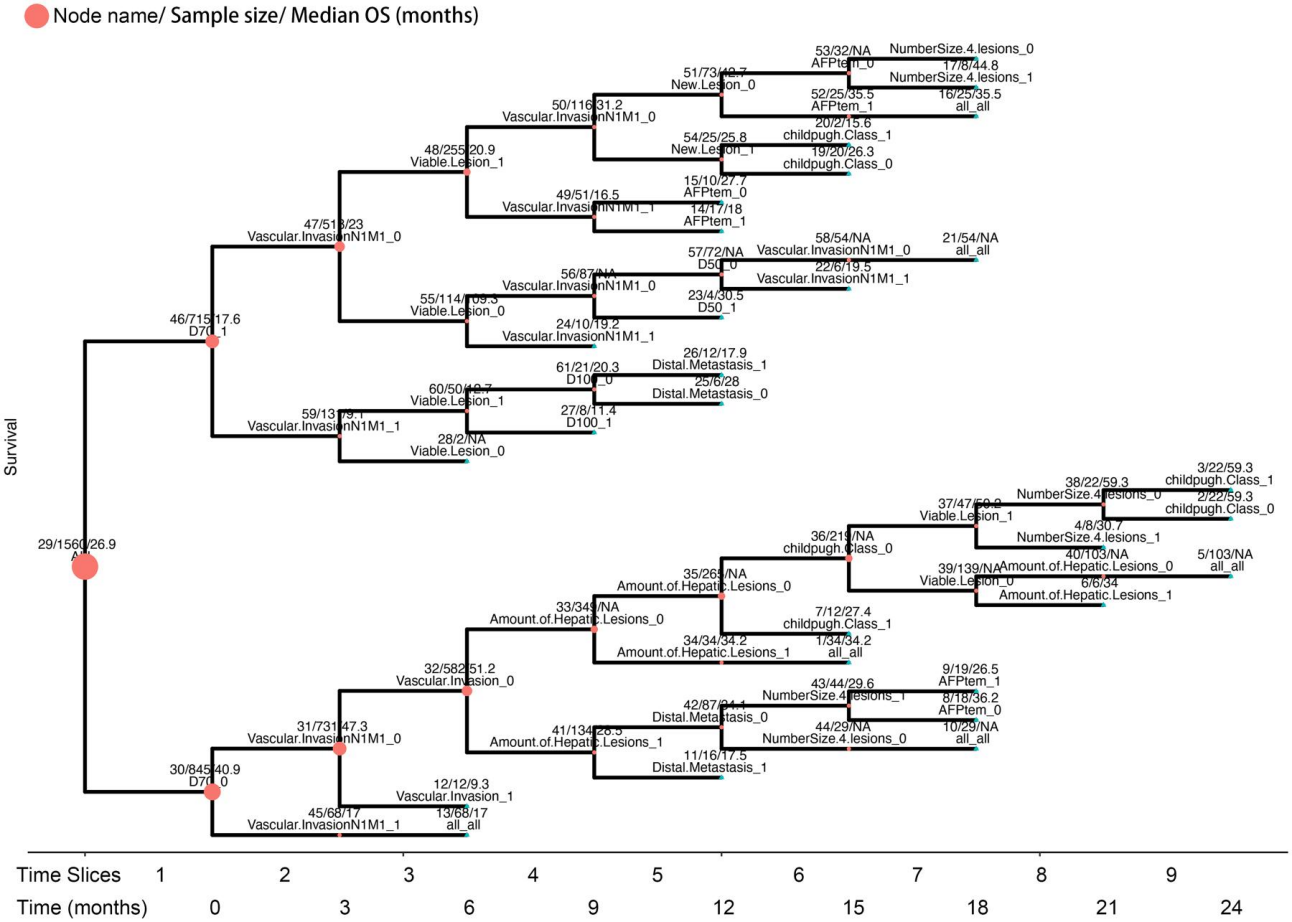
The survival path model constructed based on the training set. The numbers above each node separated by slash refers to the node name, sample size, and median OS of the patients at the specific node, respectively. The “NA” refers to the condition that median OS was not reached.

The survival path model is designed to convert the time-series data into a cascading survival map, therefore to facilitate dynamic prognosis prediction and treatment planning. Based on the derivation cohort, a survival path model containing 28 different paths was built (Fig. 3). The cutoffs of included variables are shown in Supplementary Table S1. In the survival path model, the information of bifurcation variable and associated parameters is displayed on the top of node. The first bifurcation variable was diameter of largest intrahepatic of 70 mm (D70), which divided the cohort (parent node) at time slice no. 1 into two subnodes. Most bifurcation variables appeared multiple times in the survival path model while the variable “new lesion” appeared only once in dividing node no. 50.

### 3.4 Comparison of the survival path model with conventional static machine learning models

**Figure 4.**
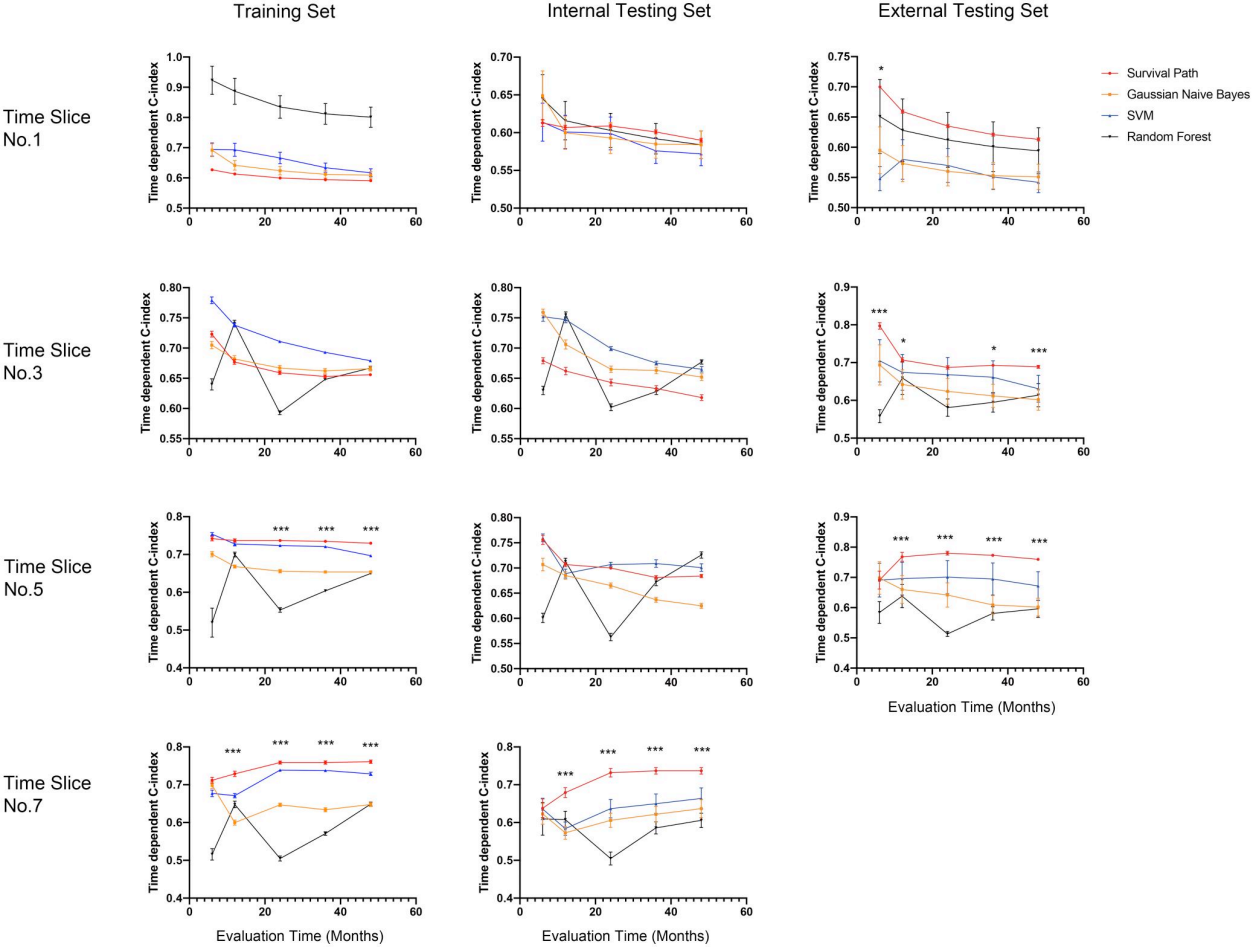
Comparison of the time-dependent *c*-index between different models. The change of time-dependent *c*-index along with different evaluation time for the four machine learning models in the training set, internal testing set, and multicentric testing set were compared. *** The SP model demonstrated superior *c*-index compared to other machine learning models, with *P*-value <.0009.

The accuracy of prognostic prediction between the survival path model and conventional static machine learning models was compared at different prediction times in training internal testing and external testing datasets (Table 2, Supplementary Table S3, Fig. 4). It is interesting to note that survival path model had no advantages at early time slices (no. 1 and no. 3). On the other hand, the SVM model demonstrated high accuracy in dynamic prognosis prediction in training and external testing sets. Although RF model demonstrated very high accuracy in time slice no. 1, its accuracy in prediction at distant evaluation times (>20 months) decreased sharply; besides, its performance in testing datasets was significantly worse than that in training set.

**Table 2.** Comparison of time-dependent *c-*index (*t*, Δ*t*) (mean ± SD) for survival path models and conventional static machine learning models.

Pred_time (time slice)	Eval_time (months)	Gaussian Naive Bayes			SVM			Random forest			Survival path		
		Train	Inter test	Exter test	Train	Inter test	Exter test	Train	Inter test	Exter test	Train	Inter test	Exter test
*t* = 1	Δ *t* = 6	0.692 ± 0.068	0.649 ± 0.104	0.595 ± 0.123	0.694 ± 0.069	0.614 ± 0.080	0.548 ± 0.063	0.923 ± 0.148	0.645 ± 0.101	0.651 ± 0.194	0.627 ± 0.011	0.613 ± 0.015	0.700 ± 0.006
	Δ *t* = 12	0.642 ± 0.050	0.600 ± 0.070	0.573 ± 0.095	0.693 ± 0.068	0.601 ± 0.070	0.580 ± 0.104	0.887 ± 0.136	0.616 ± 0.081	0.628 ± 0.164	0.613 ± 0.008	0.607 ± 0.009	0.659 ± 0.009
	Δ *t* = 24	0.624 ± 0.044	0.593 ± 0.065	0.560 ± 0.077	0.666 ± 0.059	0.599 ± 0.069	0.570 ± 0.089	0.835 ± 0.118	0.603 ± 0.072	0.612 ± 0.144	0.600 ± 0.005	0.609 ± 0.008	0.635 ± 0.009
	Δ *t* = 36	0.612 ± 0.040	0.585 ± 0.060	0.553 ± 0.069	0.634 ± 0.048	0.576 ± 0.053	0.551 ± 0.066	0.812 ± 0.109	0.592 ± 0.064	0.601 ± 0.130	0.594 ± 0.005	0.601 ± 0.008	0.621 ± 0.008
	Δ *t* = 48	0.609 ± 0.039	0.584 ± 0.059	0.551 ± 0.066	0.617 ± 0.042	0.572 ± 0.050	0.542 ± 0.054	0.801 ± 0.106	0.584 ± 0.058	0.594 ± 0.121	0.591 ± 0.005	0.590 ± 0.008	0.613 ± 0.008
*t* = 3	Δ *t* = 6	0.705 ± 0.019	0.759 ± 0.017	0.694 ± 0.168	0.779 ± 0.018	0.752 ± 0.024	0.705 ± 0.178	0.640 ± 0.029	0.630 ± 0.022	0.558 ± 0.055	0.723 ± 0.015	0.679 ± 0.016	0.797 ± 0.028
	Δ *t* = 12	0.682 ± 0.017	0.706 ± 0.024	0.642 ± 0.123	0.738 ± 0.010	0.747 ± 0.016	0.674 ± 0.150	0.741 ± 0.016	0.755 ± 0.016	0.659 ± 0.138	0.677 ± 0.012	0.662 ± 0.019	0.707 ± 0.020
	Δ *t* = 24	0.667 ± 0.014	0.665 ± 0.017	0.624 ± 0.108	0.711 ± 0.006	0.699 ± 0.011	0.668 ± 0.144	0.593 ± 0.010	0.602 ± 0.018	0.581 ± 0.073	0.659 ± 0.009	0.643 ± 0.018	0.687 ± 0.015
	Δ *t* = 36	0.662 ± 0.013	0.663 ± 0.017	0.612 ± 0.098	0.693 ± 0.006	0.675 ± 0.010	0.661 ± 0.139	0.648 ± 0.007	0.628 ± 0.015	0.595 ± 0.083	0.653 ± 0.008	0.633 ± 0.015	0.693 ± 0.010
	Δ *t* = 48	0.666 ± 0.013	0.652 ± 0.018	0.602 ± 0.089	0.679 ± 0.006	0.665 ± 0.015	0.631 ± 0.112	0.667 ± 0.010	0.677 ± 0.011	0.614 ± 0.098	0.656 ± 0.007	0.618 ± 0.015	0.689 ± 0.013
*t* = 5	Δ *t* = 6	0.701 ± 0.023	0.707 ± 0.040	0.698 ± 0.171	0.754 ± 0.013	0.760 ± 0.025	0.691 ± 0.177	0.520 ± 0.122	0.601 ± 0.030	0.584 ± 0.114	0.742 ± 0.018	0.756 ± 0.029	0.691 ± 0.094
	Δ *t* = 12	0.668 ± 0.013	0.685 ± 0.023	0.660 ± 0.146	0.728 ± 0.016	0.689 ± 0.028	0.696 ± 0.172	0.700 ± 0.021	0.713 ± 0.021	0.638 ± 0.122	0.737 ± 0.014	0.707 ± 0.014	0.768 ± 0.046
	Δ *t* = 24	0.656 ± 0.014	0.665 ± 0.016	0.642 ± 0.127	0.724 ± 0.009	0.707 ± 0.015	0.701 ± 0.173	0.553 ± 0.020	0.563 ± 0.023	0.513 ± 0.027	0.737 ± 0.011	0.700 ± 0.008	0.780 ± 0.021
	Δ *t* = 36	0.654 ± 0.009	0.637 ± 0.016	0.609 ± 0.098	0.721 ± 0.007	0.709 ± 0.024	0.695 ± 0.168	0.604 ± 0.009	0.672 ± 0.023	0.581 ± 0.070	0.735 ± 0.011	0.681 ± 0.012	0.773 ± 0.014
	Δ *t* = 48	0.654 ± 0.006	0.625 ± 0.016	0.602 ± 0.092	0.697 ± 0.009	0.701 ± 0.024	0.672 ± 0.149	0.650 ± 0.012	0.726 ± 0.020	0.596 ± 0.088	0.730 ± 0.011	0.684 ± 0.011	0.760 ± 0.015
*t* = 7	Δ *t* = 6	0.699 ± 0.028	0.623 ± 0.087	–	0.677 ± 0.028	0.636 ± 0.090	–	0.516 ± 0.048	0.610 ± 0.138	–	0.712 ± 0.025	0.638 ± 0.079	–
	Δ *t* = 12	0.600 ± 0.022	0.573 ± 0.054	–	0.671 ± 0.018	0.584 ± 0.056	–	0.648 ± 0.028	0.608 ± 0.070	–	0.729 ± 0.023	0.679 ± 0.042	–
	Δ *t* = 24	0.647 ± 0.014	0.606 ± 0.060	–	0.739 ± 0.012	0.637 ± 0.077	–	0.505 ± 0.022	0.505 ± 0.054	–	0.759 ± 0.013	0.732 ± 0.036	–
	Δ *t* = 36	0.634 ± 0.017	0.622 ± 0.066	–	0.738 ± 0.012	0.650 ± 0.082	–	0.571 ± 0.015	0.586 ± 0.051	–	0.759 ± 0.014	0.737 ± 0.028	–
	Δ *t* = 48	0.648 ± 0.017	0.637 ± 0.075	–	0.729 ± 0.014	0.664 ± 0.088	–	0.649 ± 0.017	0.606 ± 0.061	–	0.761 ± 0.014	0.737 ± 0.028	–
*t* = 9	Δ *t* = 6	0.651 ± 0.039	0.735 ± 0.051	–	0.683 ± 0.050	0.587 ± 0.100	–	0.573 ± 0.067	0.440 ± 0.202	–	0.688 ± 0.038	0.655 ± 0.046	–
	Δ *t* = 12	0.695 ± 0.026	0.629 ± 0.094	–	0.696 ± 0.040	0.625 ± 0.067	–	0.674 ± 0.036	0.607 ± 0.071	–	0.767 ± 0.022	0.697 ± 0.042	–
	Δ *t* = 24	0.683 ± 0.033	0.655 ± 0.045	–	0.777 ± 0.028	0.657 ± 0.043	–	0.533 ± 0.025	0.563 ± 0.053	–	0.782 ± 0.020	0.762 ± 0.028	–
	Δ *t* = 36	0.700 ± 0.030	0.700 ± 0.040	–	0.754 ± 0.028	0.682 ± 0.035	–	0.602 ± 0.036	0.602 ± 0.071	–	0.782 ± 0.022	0.762 ± 0.028	–
	Δ *t* = 48	0.654 ± 0.021	0.698 ± 0.038	–	0.770 ± 0.037	0.679 ± 0.032	–	0.685 ± 0.020	0.566 ± 0.066	–	0.782 ± 0.021	0.762 ± 0.028	–

The survival path model had superior or non-inferior performance in prognosis prediction compared to GNB and RF models at late time slices (no. 5 and no. 7). Although in the internal testing set, the SVM model had superior performance compared to the survival path model for prediction of specific evaluation time at time slice no. 5, the advantages did not appear in the external testing cohort. The survival path model had higher time-dependent *c*-index over all conventional ML models since time slice no. 3 in the external testing cohort.

## 4 Discussion

In this study, we compared the performance on dynamic prognosis prediction between the survival path model and conventional static machine learning models in a large cohort of patients with HCC. Our study found that although conventional ML model has higher predictive ability for patient at initial admission when the prediction time comes to the 12 months or even later since the initial diagnosis, the survival path model significantly outperforms the conventional ML models in long-term prognosis prediction. Moreover, the generalization ability of survival path model is remarkable as it demonstrated much better performance than conventional static ML models in multicentric testing dataset. These results suggested that although there are many machine learning models available in building static prediction models, the survival path model has unique advantages in dynamic prediction through utilizing time-series data of HCC patients with key features identified. Moreover, the survival path models offer superior visualization capabilities compared to SVM, RF and GNB, and therefore may be easier to be accepted by oncologists.

BCLC Stage B HCC represents a heterogeneous group of disease, of which the status changed rapidly during treatment (Kim *et al.* 2023). As depicted in Fig. 2, even after dimensionality reduction with PCA, the key covariates cannot distinguish different survival endpoints, suggesting that data from single time point cannot accurately predict long-term prognosis of HCC and a comprehensive analysis of time-series data may contribute to more accurate prediction.

In the field of cancer prediction, GNB (Kaviarasi and Gandhi 2019), RF (Li *et al.* 2020a), and SVMs (Jiang *et al.* 2018) are three commonly used machine learning modeling methods (Swanson *et al.* 2023). In the majority of previous studies, static ML models were built and applied to predict survival outcomes based on baseline data from the initial diagnosis (Sun *et al.* 2023). Different ML algorithms have different features. The GNB model is simple and computationally efficient, suitable for handling high-dimensional data, but its accuracy may be affected by its assumption of feature independence. RF can handle high-dimensional and noisy data, is robust and less prone to overfitting, but has higher model complexity and longer training times compared to Bayesian models. SVMs perform well on small datasets, can address high-dimensional problems, and handle non-linear issues through different kernel functions, but have low efficiency in processing large-scale data (Lai *et al.* 2020).

During the internal testing, there appears to be no significant advantages of the survival path models over traditional static ML models when predicting survival at time slices 1, 3, and 5, but a notable superiority was at time slice no. 7. Moreover, we found that the accuracy of the different static ML models gradually decreases over time. This can be best exemplified in the performance of GNB, which present a gradual decline in prognostic accuracy with the advancement of prediction time and evaluation time. On the other hand, the SVM model also only uses data at initial diagnosis for training and performs excellently on the training set, but underperforms in internal and external testing cohorts, suggesting that potential overfitting exists. The survival path model, capable of utilizing the time series in modeling, performs well in both the training set and internal testing cohort, presents relatively stable predictive accuracy across different time slices. Furthermore, the survival path model shows significantly better performance in the multicentric external testing cohort compared to traditional static machine learning models, indicating it is an effective and stable method for dynamic prognosis prediction of HCC. These phenomena also offered us an insight that utilization of time-series data is of vital importance in modeling to achieve dynamic prognostication of HCC.

During our analysis, it is intriguing to find that the RF model presents an unstable pattern of predictive accuracy with the advancement of prediction time and evaluation time. One possible explanation may be that the RF model relies much on certain key features identified at the initial diagnosis, and omitted other contributing factors that may also played a role in prognosticating (Supplementary Table S4). Only a model that comprehensively incorporated the majority of key variables can achieve relatively stable predictive ability across different prediction and evaluation times for patients with HCC.

As a novel modeling algorithm, another advantage of the survival path model is its strong visualization capability. Traditional machine learning models, including RF, SVMs, and Naive Bayes, have weak visualization capabilities (Li *et al.* 2020b), while popular deep learning approaches are almost all black boxes models, making them difficult to read and understand by researchers (Hosny *et al.* 2018, Lee *et al.* 2020). In the survival path model, each categorical variable is processed in a binary manner, allowing for model visualization while balancing the efficiency of data usability. Additionally, the survival path model supports dynamic treatment decisions. When patients reach a critical node, we can quickly identify key variables at the bifurcation point, thereby assisting in developing personalized treatment strategies. The SurvivalPath R package also includes functions for comparing different treatment plans, facilitating exploring potential beneficial treatment options for patients (Shen *et al.* 2023).

In recent years, many innovative models have emerged that use longitudinal clinical data to predict the prognosis of cancer patients. Joint modeling is currently the mainstream modeling strategy. Li *et al.* (2023) used multivariate principal components analysis (MFPCA) to characterize the changing patterns of the multivariate longitudinal processes, and employed random survival forest as a survival submodel to achieve personalized dynamic prediction for CRC patients after surgery. In terms of the field of prostate cancer, a considerable amount of studies explored joint modeling accounting for two processes, the longitudinal model (prostate-specific antigen levels), and the time-to-event process (clinical failure), for achieving dynamic prediction of recurrence after surgery (Parr *et al.* 2022). Meanwhile, as joint models may be limited by parametric assumptions in both the longitudinal and survival submodels, machine learning through neural networks emerged as a new tool in modeling of longitudinal data for survival prediction. The Dynamic Deephit, based on RNN and attention mechanism, was proposed and has demonstrated to have advantages in active surveillance for prostate cancer compared to Cox regression model (Lee *et al.* 2022). A comparison between the survival path model and these machine learning methodologies in dynamic prognosis prediction for HCC patients is needed in the future.

Our study has several limitations. First, it is a retrospective study using dataset of single center. Second, the time-series data after patients start to lose surveillance during specific time slice were not included in the analysis, which may limit the scope of application of the conclusion. Thirdly, last observation carry forward (LOCF) imputation was utilized for patients who achieved cancer free status (CFS) after treatment and maintained CFS during the late time slices. Potential bias may be introduced. However, the impact was trivial as only a small proportion (<2%) of patients had such imputation. Therefore, to further confirm the value of this methodology in HCC, a large-scale multi-institutional prospective study for both modeling and validation is needed.

## 5 Conclusion

Although conventional static machine learning models had advantages over survival path model in dynamic prognostication of HCC patients at early prediction times, the survival path model outperforms the conventional ML models in long-term prognosis prediction (evaluation time ≥20 months) at late prediction times (≥12 months). The SP models, given its features of easy to visualize, use, and understand, have the potential to enter the clinic in the near future.

## Supplementary Material

vbaf027_Supplementary_Data

## Data Availability

Data are available from the corresponding author upon reasonable request.
