# Supplementary material for: Survival path model outperforms conventional static machine learning models in long-term dynamic prognosis prediction for patients with intermediate stage hepatocellular carcinoma
Source: Bioinform Adv. 2025 Feb 17;5(1):vbaf027. doi: 10.1093/bioadv/vbaf027 (PMC11978388; doi:10.1093/bioadv/vbaf027)
Supplement: vbaf027_Supplementary_Data [file vbaf027_supplementary_data.docx]

**Supplement to “Survival path model outperforms conventional static machine learning models in long-term dynamic prognosis prediction for patients with intermediate stage hepatocellular carcinoma”**

| Categories | Survival Path with curated variable | | |
| --- | --- | --- | --- |
|  | Variable | Cutoff | Coding |
| **Imaging examination** | Diameter of largest intrahepatic lesion (mm) | ≤82/ >82 | 0/1 |
|  | Diameter of largest intrahepatic of 70 mm (D70) | Below/Above | 0/1 |
|  | D50 | Below/Above | 0/1 |
|  | D70 | Below/Above | 0/1 |
|  | Number and size of hepatic lesions / ≥4 lesions | ≤1 lesion/2–3 lesions, D ≤ 30 mm vs. >3 lesions/2–3 lesions, D > 30 mm | 0/1 |
|  | Vascular Invasion | Absent/Present | 0/1 |
|  | Distant Metastasis | Absent/Present | 0/1 |
|  | Vascular Invasion/N1/M1 | Absent/Present | 0/1 |
| **Change of Lesions** | New lesion | Absent/Present | 0/1 |
|  | Viable Lesion | Absent/Present | 0/1 |
| **Laboratory Tests** | Child Pugh Class | class A vs. Class B/C;  class A/B vs. Class C | 0/1 |
|  | Serum AFP ng/ml (AFPtem) | <13.94/ ≥13.94 | 0/1 |
|  | Serum AFP level of 400 ng/ml (AFP 400) | <400/ ≥400 | 0/1 |
|  | Serum AFP level of 200 ng/ml (AFP 200) | <200/ ≥200 | 0/1 |

Table S1. Variables and cutoffs of the constructed survival path models

Table S2. The variables and parameters in building the conventional machine learning models

| Variables | Gaussian Naive Bayes | SVM | Random Forest |
| --- | --- | --- | --- |
| **Age** | Continuous variable | Continuous variable | Continuous variable |
| **Laboratory Tests** |  |  |  |
| Serum AFP level | Continuous variable | Continuous variable, <200 vs. ≥200; <400 vs. ≥400 | Continuous variable |
| Child Pugh Class | Ordinal variable (1,2,3) | Ordinal variable, Class B/C vs. class A; class C vs. class A/B | / |
| ALB level | / | / | Continuous variable |
| TBIL level | / | / | Continuous variable |
| PT | / | / | Continuous variable |
| HBV infection | / | / | Present/Absent |
| **Imaging examination** |  |  |  |
| Diameter of largest intrahepatic lesion (mm) | Continuous variable | Continuous variable; ≤50 vs. >50; ≤70 vs. >70; ≤100 vs. >100 | Continuous variable |
| Number of hepatic lesions | Continuous variable | Continuous variable, <4 lesions vs. ≥4 lesions | Continuous variable |
| Vascular invasion | With vs. without | With vs. without | With vs. without |
| Distant metastasis | With vs. without | With vs. without | With vs. without |
| Lymph Node metastasis | With vs. without | With vs. without | With vs. without |
| Lymph Node or distant metastasis/ Vascular Invasion (NMV) | / | With vs. without | / |
| Number and size of hepatic lesions | / | ≤1 lesion/2–3 lesions, D ≤ 30 mm vs. >3 lesions/2–3 lesions, D > 30 mm | / |
| Number and size of hepatic lesions, NMV | / | ≤1 lesion/2–3 lesions, D ≤ 30 mm, without NMV vs. >3 lesions/2–3 lesions, D > 30 mm/NMV | / |
| Change of lesions | / | With viable lesion vs. without viable lesion; With new lesion/ without new lesion | With new lesion/ without new lesion |
| Ascites | / | / | Present/Absent; Massive/not Massive |
| **Model parameters** | Priors = None,  Var_smoothing = 1e^-09^ | kernel="rbf",  gamma="auto",  cache_size=5000,  probability=True | n_estimators="10",  Criterion ="gini",  Min_samples_split =2,  Min_samples_leaf = 1,  Max_depth ="None" |

Note: The source data in building survival path models and conventional machine learning models were the same.

Table S3. Comparison of time-dependent C index (t, ∆t) (mean±SD) for between different models from time slice no. 1 to no.9

| Pred_time (time slice) | Eval_time (months) | Gaussian Naive Bayes | | | SVM | | | Random Forest | | | Survival Path | | |
| --- | --- | --- | --- | --- | --- | --- | --- | --- | --- | --- | --- | --- | --- |
|  |  | Train | Inter test | Exter test | Train | Inter test | Exter test | Train | Inter test | Exter test | Train | Inter test | Exter test |
| t=1 | ∆t=6 | 0.692±0.068 | 0.649±0.104 | 0.595±0.123 | 0.694±0.069 | 0.614±0.080 | 0.548±0.063 | 0.923±0.148 | 0.645±0.101 | 0.651±0.194 | 0.627±0.011 | 0.613±0.015 | 0.700±0.006 |
|  | ∆t=12 | 0.642±0.050 | 0.600±0.070 | 0.573±0.095 | 0.693±0.068 | 0.601±0.070 | 0.580±0.104 | 0.887±0.136 | 0.616±0.081 | 0.628±0.164 | 0.613±0.008 | 0.607±0.009 | 0.659±0.009 |
|  | ∆t=24 | 0.624±0.044 | 0.593±0.065 | 0.560±0.077 | 0.666±0.059 | 0.599±0.069 | 0.570±0.089 | 0.835±0.118 | 0.603±0.072 | 0.612±0.144 | 0.600±0.005 | 0.609±0.008 | 0.635±0.009 |
|  | ∆t=36 | 0.612±0.040 | 0.585±0.060 | 0.553±0.069 | 0.634±0.048 | 0.576±0.053 | 0.551±0.066 | 0.812±0.109 | 0.592±0.064 | 0.601±0.130 | 0.594±0.005 | 0.601±0.008 | 0.621±0.008 |
|  | ∆t=48 | 0.609±0.039 | 0.584±0.059 | 0.551±0.066 | 0.617±0.042 | 0.572±0.050 | 0.542±0.054 | 0.801±0.106 | 0.584±0.058 | 0.594±0.121 | 0.591±0.005 | 0.590±0.008 | 0.613±0.008 |
| t=2 | ∆t=6 | 0.740±0.012 | 0.732±0.019 | 0.811±0.023 | 0.742±0.014 | 0.786±0.014 | 0.846±0.018 | 0.711±0.013 | 0.675±0.019 | 0.707±0.032 | 0.678±0.010 | 0.737±0.015 | 0.826±0.017 |
|  | ∆t=12 | 0.693±0.006 | 0.679±0.016 | 0.724±0.017 | 0.711±0.005 | 0.721±0.016 | 0.723±0.013 | 0.775±0.007 | 0.696±0.013 | 0.748±0.013 | 0.656±0.009 | 0.692±0.011 | 0.719±0.017 |
|  | ∆t=24 | 0.672±0.006 | 0.662±0.015 | 0.687±0.013 | 0.679±0.007 | 0.668±0.012 | 0.708±0.010 | 0.682±0.007 | 0.637±0.010 | 0.644±0.019 | 0.635±0.008 | 0.670±0.007 | 0.684±0.014 |
|  | ∆t=36 | 0.660±0.007 | 0.653±0.014 | 0.689±0.012 | 0.654±0.006 | 0.638±0.011 | 0.684±0.013 | 0.701±0.006 | 0.647±0.008 | 0.695±0.010 | 0.626±0.007 | 0.656±0.006 | 0.668±0.014 |
|  | ∆t=48 | 0.657±0.007 | 0.646±0.012 | 0.681±0.013 | 0.632±0.007 | 0.630±0.012 | 0.649±0.012 | 0.700±0.006 | 0.647±0.010 | 0.675±0.009 | 0.622±0.006 | 0.638±0.006 | 0.645±0.012 |
| t=3 | ∆t=6 | 0.705±0.019 | 0.759±0.017 | 0.694±0.168 | 0.779±0.018 | 0.752±0.024 | 0.705±0.178 | 0.640±0.029 | 0.630±0.022 | 0.558±0.055 | 0.723±0.015 | 0.679±0.016 | 0.797±0.028 |
|  | ∆t=12 | 0.682±0.017 | 0.706±0.024 | 0.642±0.123 | 0.738±0.010 | 0.747±0.016 | 0.674±0.150 | 0.741±0.016 | 0.755±0.016 | 0.659±0.138 | 0.677±0.012 | 0.662±0.019 | 0.707±0.020 |
|  | ∆t=24 | 0.667±0.014 | 0.665±0.017 | 0.624±0.108 | 0.711±0.006 | 0.699±0.011 | 0.668±0.144 | 0.593±0.010 | 0.602±0.018 | 0.581±0.073 | 0.659±0.009 | 0.643±0.018 | 0.687±0.015 |
|  | ∆t=36 | 0.662±0.013 | 0.663±0.017 | 0.612±0.098 | 0.693±0.006 | 0.675±0.010 | 0.661±0.139 | 0.648±0.007 | 0.628±0.015 | 0.595±0.083 | 0.653±0.008 | 0.633±0.015 | 0.693±0.010 |
|  | ∆t=48 | 0.666±0.013 | 0.652±0.018 | 0.602±0.089 | 0.679±0.006 | 0.665±0.015 | 0.631±0.112 | 0.667±0.010 | 0.677±0.011 | 0.614±0.098 | 0.656±0.007 | 0.618±0.015 | 0.689±0.013 |
| t=4 | ∆t=6 | 0.727±0.020 | 0.739±0.028 | 0.500±0.000 | 0.721±0.017 | 0.796±0.036 | 0.500±0.000 | 0.593±0.060 | 0.681±0.038 | 0.500±0.000 | 0.717±0.016 | 0.752±0.024 | 0.728±0.038 |
|  | ∆t=12 | 0.667±0.017 | 0.721±0.014 | 0.500±0.000 | 0.710±0.014 | 0.734±0.027 | 0.500±0.000 | 0.714±0.015 | 0.774±0.026 | 0.500±0.000 | 0.698±0.015 | 0.711±0.023 | 0.725±0.030 |
|  | ∆t=24 | 0.671±0.009 | 0.683±0.013 | 0.500±0.000 | 0.713±0.010 | 0.714±0.015 | 0.500±0.000 | 0.574±0.013 | 0.639±0.014 | 0.500±0.000 | 0.704±0.009 | 0.680±0.022 | 0.748±0.021 |
|  | ∆t=36 | 0.666±0.010 | 0.701±0.011 | 0.500±0.000 | 0.705±0.010 | 0.723±0.019 | 0.500±0.000 | 0.633±0.012 | 0.649±0.020 | 0.500±0.000 | 0.695±0.007 | 0.677±0.129 | 0.734±0.018 |
|  | ∆t=48 | 0.657±0.010 | 0.695±0.014 | 0.500±0.000 | 0.688±0.010 | 0.700±0.023 | 0.500±0.000 | 0.649±0.011 | 0.698±0.027 | 0.500±0.000 | 0.696±0.006 | 0.679±0.012 | 0.723±0.016 |
| t=5 | ∆t=6 | 0.701±0.023 | 0.707±0.040 | 0.698±0.171 | 0.754±0.013 | 0.760±0.025 | 0.691±0.177 | 0.520±0.122 | 0.601±0.030 | 0.584±0.114 | 0.742±0.018 | 0.756±0.029 | 0.691±0.094 |
|  | ∆t=12 | 0.668±0.013 | 0.685±0.023 | 0.660±0.146 | 0.728±0.016 | 0.689±0.028 | 0.696±0.172 | 0.700±0.021 | 0.713±0.021 | 0.638±0.122 | 0.737±0.014 | 0.707±0.014 | 0.768±0.046 |
|  | ∆t=24 | 0.656±0.014 | 0.665±0.016 | 0.642±0.127 | 0.724±0.009 | 0.707±0.015 | 0.701±0.173 | 0.553±0.020 | 0.563±0.023 | 0.513±0.027 | 0.737±0.011 | 0.700±0.008 | 0.780±0.021 |
|  | ∆t=36 | 0.654±0.009 | 0.637±0.016 | 0.609±0.098 | 0.721±0.007 | 0.709±0.024 | 0.695±0.168 | 0.604±0.009 | 0.672±0.023 | 0.581±0.070 | 0.735±0.011 | 0.681±0.012 | 0.773±0.014 |
|  | ∆t=48 | 0.654±0.006 | 0.625±0.016 | 0.602±0.092 | 0.697±0.009 | 0.701±0.024 | 0.672±0.149 | 0.650±0.012 | 0.726±0.020 | 0.596±0.088 | 0.730±0.011 | 0.684±0.011 | 0.760±0.015 |
| t=6 | ∆t=6 | 0.678±0.018 | 0.653±0.047 | 0.500±0.000 | 0.740±0.024 | 0.792±0.040 | 0.500±0.000 | 0.507±0.079 | 0.695±0.058 | 0.500±0.000 | 0.727±0.022 | 0.717±0.040 | 0.768±0.120 |
|  | ∆t=12 | 0.637±0.017 | 0.645±0.037 | 0.500±0.000 | 0.664±0.015 | 0.699±0.034 | 0.500±0.000 | 0.683±0.016 | 0.731±0.033 | 0.500±0.000 | 0.704±0.011 | 0.682±0.032 | 0.789±0.056 |
|  | ∆t=24 | 0.674±0.017 | 0.642±0.025 | 0.500±0.000 | 0.717±0.012 | 0.703±0.014 | 0.500±0.000 | 0.531±0.027 | 0.590±0.028 | 0.500±0.000 | 0.728±0.009 | 0.693±0.022 | 0.767±0.049 |
|  | ∆t=36 | 0.668±0.015 | 0.600±0.034 | 0.500±0.000 | 0.718±0.012 | 0.675±0.015 | 0.500±0.000 | 0.598±0.012 | 0.623±0.025 | 0.500±0.000 | 0.728±0.009 | 0.691±0.021 | 0.762±0.040 |
|  | ∆t=48 | 0.671±0.016 | 0.605±0.031 | 0.500±0.000 | 0.724±0.015 | 0.676±0.014 | 0.500±0.000 | 0.665±0.012 | 0.676±0.016 | 0.500±0.000 | 0.725±0.009 | 0.691±0.021 | 0.756±0.035 |
| t=7 | ∆t=6 | 0.699±0.028 | 0.623±0.087 | - | 0.677±0.028 | 0.636±0.090 | - | 0.516±0.048 | 0.610±0.138 | - | 0.712±0.025 | 0.638±0.079 | - |
|  | ∆t=12 | 0.600±0.022 | 0.573±0.054 | - | 0.671±0.018 | 0.584±0.056 | - | 0.648±0.028 | 0.608±0.070 | - | 0.729±0.023 | 0.679±0.042 | - |
|  | ∆t=24 | 0.647±0.014 | 0.606±0.060 | - | 0.739±0.012 | 0.637±0.077 | - | 0.505±0.022 | 0.505±0.054 | - | 0.759±0.013 | 0.732±0.036 | - |
|  | ∆t=36 | 0.634±0.017 | 0.622±0.066 | - | 0.738±0.012 | 0.650±0.082 | - | 0.571±0.015 | 0.586±0.051 | - | 0.759±0.014 | 0.737±0.028 | - |
|  | ∆t=48 | 0.648±0.017 | 0.637±0.075 | - | 0.729±0.014 | 0.664±0.088 | - | 0.649±0.017 | 0.606±0.061 | - | 0.761±0.014 | 0.737±0.028 | - |
| t=8 | ∆t=6 | 0.655±0.033 | 0.624±0.051 | - | 0.604±0.034 | 0.501±0.071 | - | 0.525±0.034 | 0.518±0.045 | - | 0.686±0.022 | 0.624±0.083 | - |
|  | ∆t=12 | 0.625±0.036 | 0.638±0.034 | - | 0.647±0.019 | 0.613±0.048 | - | 0.629±0.019 | 0.623±0.055 | - | 0.720±0.023 | 0.720±0.066 | - |
|  | ∆t=24 | 0.646±0.027 | 0.727±0.022 | - | 0.764±0.017 | 0.707±0.025 | - | 0.513±0.016 | 0.530±0.040 | - | 0.774±0.020 | 0.747±0.049 | - |
|  | ∆t=36 | 0.664±0.024 | 0.697±0.021 | - | 0.749±0.013 | 0.672±0.035 | - | 0.578±0.022 | 0.575±0.042 | - | 0.786±0.015 | 0.748±0.046 | - |
|  | ∆t=48 | 0.644±0.026 | 0.702±0.022 | - | 0.732±0.023 | 0.686±0.033 | - | 0.667±0.017 | 0.634±0.042 | - | 0.793±0.012 | 0.748±0.046 | - |
| t=9 | ∆t=6 | 0.651±0.039 | 0.735±0.051 | - | 0.683±0.050 | 0.587±0.100 | - | 0.573±0.067 | 0.440±0.202 | - | 0.688±0.038 | 0.655±0.046 | - |
|  | ∆t=12 | 0.695±0.026 | 0.629±0.094 | - | 0.696±0.040 | 0.625±0.067 | - | 0.674±0.036 | 0.607±0.071 | - | 0.767±0.022 | 0.697±0.042 | - |
|  | ∆t=24 | 0.683±0.033 | 0.655±0.045 | - | 0.777±0.028 | 0.657±0.043 | - | 0.533±0.025 | 0.563±0.053 | - | 0.782±0.020 | 0.762±0.028 | - |
|  | ∆t=36 | 0.700±0.030 | 0.700±0.040 | - | 0.754±0.028 | 0.682±0.035 | - | 0.602±0.036 | 0.602±0.071 | - | 0.782±0.022 | 0.762±0.028 | - |
|  | ∆t=48 | 0.654±0.021 | 0.698±0.038 | - | 0.770±0.037 | 0.679±0.032 | - | 0.685±0.020 | 0.566±0.066 | - | 0.782±0.021 | 0.762±0.028 | - |

**Table S4. The feature importance of variables in Random Forest Model in prediction survival of HCC patients.**

| Categories | 6 months | 12 months | 24 months | 36 months | 48 months |
| --- | --- | --- | --- | --- | --- |
| Age | 0.1306 | 0.1221 | 0.1226 | 0.1244 | 0.1273 |
| Amount of Hepatic Lesions | 0.0337 | 0.0378 | 0.0531 | 0.0494 | 0.0480 |
| Largest Diameter of Hepatic Lesions (mm) | 0.1798 | 0.1814 | 0.1836 | 0.1799 | 0.1784 |
| New Lesion | 0.0031 | 0.0032 | 0.0039 | 0.0039 | 0.0035 |
| Vascular Invasion | 0.0031 | 0.0077 | 0.0049 | 0.0039 | 0.0036 |
| Local Lymph Node Metastasis | 0.0054 | 0.0094 | 0.0097 | 0.0082 | 0.0083 |
| Distal Metastasis | 0.0055 | 0.0035 | 0.0031 | 0.0030 | 0.0033 |
| Massive Ascites | 0.0029 | 0.0005 | 0.0002 | 0.0001 | 0.0001 |
| Moderate or Mild Ascites | 0.0035 | 0.0028 | 0.0030 | 0.0030 | 0.0030 |
| AFP | 0.1627 | 0.1681 | 0.1644 | 0.1580 | 0.1541 |
| ALB | 0.1567 | 0.1618 | 0.1559 | 0.1652 | 0.1675 |
| TBLT | 0.1553 | 0.1461 | 0.1450 | 0.1480 | 0.1483 |
| PT | 0.1263 | 0.1248 | 0.1238 | 0.1275 | 0.1278 |
| Child Pugh Score | 0.0242 | 0.0247 | 0.0213 | 0.0197 | 0.0207 |
